# Genome-wide association study of the response of patients with diabetic macular edema to intravitreal Anti-VEGF injection

**DOI:** 10.1038/s41598-022-26048-7

**Published:** 2022-12-29

**Authors:** Eun Hee Hong, Hoseok Yeom, Hyo Seon Yu, Jong Eun Park, Yong Un Shin, So-Young Bang, Heeyoon Cho

**Affiliations:** 1 Department of Ophthalmology, Hanyang University College of Medicine, Seoul, Republic of Korea; 2 Department of Ophthalmology, Asan Medical Center, University of Ulsan College of Medicine, Seoul, Republic of Korea; 3 Department of Laboratory Medicine, Hanyang University Guri Hospital, Hanyang University College of Medicine, Guri, Republic of Korea; 4 Department of Rheumatology, Hanyang University Hospital for Rheumatic Diseases, Seoul, Republic of Korea

**Keywords:** Genome-wide association studies, Retinal diseases, Type 2 diabetes, Prognostic markers

## Abstract

Diabetic macular edema (DME), a complication of diabetes mellitus, is a leading cause of adult-onset blindness worldwide. Recently, intravitreal anti-VEGF injection has been used as a first-line treatment. This study analyzed the association between the genetic profile of patients with DME and their response to treatment. Intravitreal anti-VEGF injections were administered monthly for three months to Korean patients diagnosed with DME, who were classified into two groups depending on whether they responded to anti-VEGF therapy or showed recurrence within six months. Peripheral blood samples were used for genetic analyses. Genome-wide association analysis results sowed that the genes *DIRC3* on chromosome 2 (rs16857280, *p* = 1.2 × 10^–6^), *SLCO3A1* on chromosome 15 (rs12899055, *p* = 2.5 × 10^–6^), and *RAB2A* on chromosome 8 (rs2272620, *p* = 4.6 × 10^–6^) were associated with treatment response to intravitreal anti-VEGF injection. *SLC35F1*, *TMEM132D*, *KIAA0368*, *HPCAL1*, *IGF2BP3*, *SPN2S*, *COL23A1*, and *CREB5* were also related to treatment response (*p* < 5.0 × 10^–5^). Using the KEGG pathway analysis, *RAB2A* and *CREB5* were found to be associated with AMPK signaling related to VEGF (*p* = 0.018). The identified genetic biomarkers can elucidate the factors affecting patient response to intravitreal anti-VEGF injection and help select appropriate therapeutic strategy.

## Introduction

Diabetic retinopathy (DR) is one of the leading causes of vision loss worldwide^1^. Diabetic macular edema (DME) is the most common cause of vision loss in patients with type 2 diabetes^2^. DME can occur at any stage of DR. There is neuronal loss, local inflammation, and pericyte loss, leading to microaneurysms that can leak fluid into the retina^3,4^. When fluid leaks into the macular area, subretinal and intraretinal fluid accumulate in the inner and outer reticular layers, leading to the swelling or thickening of the macula and development of DME^5,6^.

Based on the Early Treatment Diabetic Retinopathy Study (ETDRS), macular laser photocoagulation has been used at the point of leakage to treat non-center-involved DME and has been the gold standard for managing DME for many years. Refractory DME has been treated by removing tractional components via vitrectomy, increasing the oxygen level of the vitreous, and increasing the clearance rates of inflammatory cytokines and vascular endothelial growth factor (VEGF). Recently, VEGF has been considered to play a key role in DME, and intravitreal anti-VEGF injections have been used as the primary treatment. This treatment has also been proven to induce the regression of new vessels and improve the stage of DR^7^. Despite the widespread use of various anti-VEGF agents, it has been reported that about 30% of the patients do not respond to them. This is because complex mechanisms, such as inflammation, affect the development of DME^8,9^.

Current treatments are not always effective in patients with DME. Disease progression does not correlate with the patient’s lifestyle, and the response to anti-VEGF treatment also may vary^10^. The pathophysiology may differ depending on genetic variations. The complex mechanisms involved in the disease progression need to be analyzed further. Genome-wide association studies (GWAS) are an efficient method for identifying genetic variants associated with complex diseases, including DR. Several GWAS for DR-related phenotypes have been reported recently^10^. GWAS have also been performed using cohorts of patients with DME. Many of these studies claim to have found genetic associations; however, there are few studies related to the therapeutic effect.

The purpose of this study was to analyze the genetic association in patients with DME according to their response to the primary treatment, intravitreal anti-VEGF injection. For the evaluation, Korean patients with underlying diabetes mellitus were classified into two groups; a good responder group in which the macula was maintained without recurrence or edema, and a poor responder group.

## Method

This prospective study included patients with defined ophthalmological status who received intravitreal injections to treat non-proliferative diabetic retinopathy (NPDR) with DME from a single physician (H Cho) at the Hanyang University Guri Hospital between May 2016 and August 2017. This study was approved by the Institutional Review Board of Hanyang University Guri Hospital (IRB FILE No. 2016–01-007) and adhered to the tenets of the Declaration of Helsinki. All participants provided written informed consent to participate in the study.

### Study subjects

The inclusion criteria were treatment-naïve patients aged 18 years or older, presence of diabetes mellitus type 2, treatment with oral anti-hyperglycemic agents or insulin, and presence of center-involved DME with central subfield thickness (CST) measuring over 300 μm. The medical histories of all patients were reviewed, and those diagnosed with hypertension, dyslipidemia, or nephropathy and being treated or adequately controlled through on-going treatments were identified. Patients with one or more of the following criteria were excluded from the study: history of ocular surgery within the past 6 months, such as cataract extraction, active proliferative diabetic retinopathy (PDR), history of vitreous hemorrhage or preretinal hemorrhage presumably linked to PDR; previous treatment for DR, such as panretinal photocoagulation, intravitreal injection, or vitrectomy; or any history of coexisting pathology that could cause macular edema, such as retinal vascular disease, retinal dystrophies, and degeneration, ocular inflammatory disease, epiretinal membrane or vitreo-macular traction, uncontrolled hypertension (systolic blood pressure > 160 mmHg or diastolic blood pressure > 100 mmHg), uncontrolled diabetes mellitus (HbA1c > 10.0%), end-stage renal disease, heart failure, anemia, autoimmune disease, or infection.

All participants underwent standard ophthalmologic examination, including best-corrected visual acuity, slit lamp biomicroscopy, optical coherence tomography (swept source OCT, Topcon DRI OCT-1 Atlantis; Topcon, Inc., Tokyo, Japan), and wide-field fluorescein angiography (FA; Optos, Dunfermline, Scotland). The scanning protocol consisted of fast macular thickness maps as well as a 3D OCT 12.0 × 9.0 mm scan.

**Figure 1:**
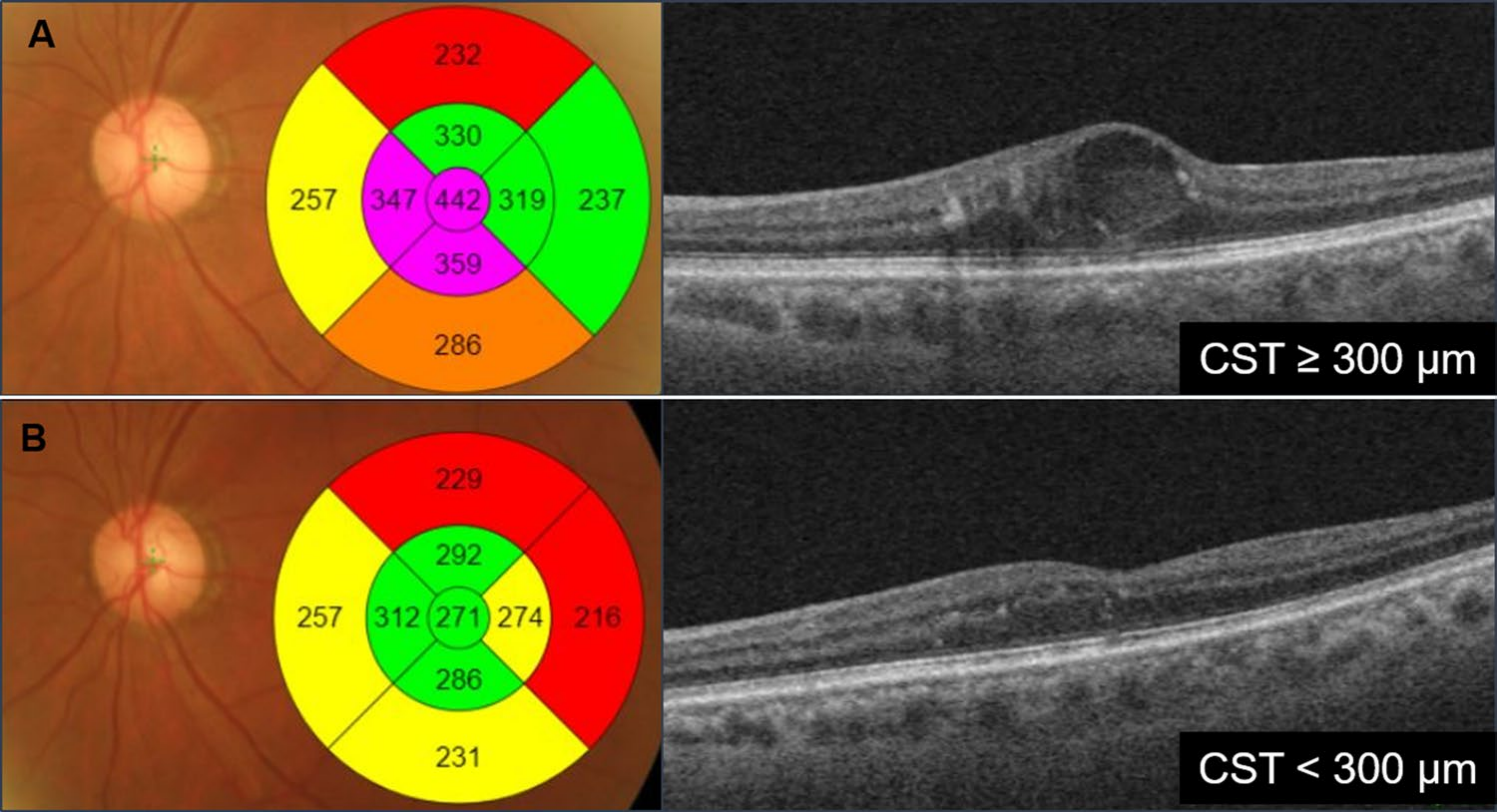
Changes in CST after intravitreal anti-VEGF injection. (**A**) Before, and (**B**) after 1 month. CST: central subfield thickness.

Macular thickness was measured using the built-in OCT software. DME was defined as the thickening of the retina (CST ≥ 300 μm, Fig. 1a)^11^. Bevacizumab (Avastin, Genentech) was administered intravitreally at the baseline visit, and at 30 ± 3 days and 60 ± 3 days from the baseline visit. Treatment response was defined as CST < 300 μm after intravitreal injection (Fig. 1b). The patients with DME were classified as good or poor responders by an experienced ophthalmologist (H Cho) after the last intravitreal anti-VEGF injection. The good responder group was defined as those who achieved CST < 300 μm for up to 6 months after the last injection. Refractory DME was defined as patients who did not achieve CST < 300 μm even after treatment. The poor responder group included patients with refractory or recurrent DME, defined as CST ≥ 300 μm within 6 months^12–14^.

### Genome-wide SNP genotyping and quality controls

Prior to the first intravitreal injection, peripheral blood samples were obtained with informed consent. The Illumina OmniExpressExome-8 BeadChip was used to conduct genome-wide genotyping analysis, according to the Infinium HD protocol (Illumina, CA, USA). 200 ng of genomic DNA from each sample was whole-genome amplified, fragmented, precipitated, and resuspended in the appropriate hybridization buffer. After hybridization of the denatured samples onto the prepared BeadChips, the BeadChips were extended using a single labeled base. Then they were stained and imaged on the Illumina Bead Array Reader to obtain normalized bead intensity data for each sample. The data were loaded into the Illumina GenomeStudio software (v2011.1), which converted the fluorescence intensities into single nucleotide polymorphism (SNP) genotypes. For 960,912 markers on the chip, SNPs with a call rate lower than 95%, a minor allele frequency < 1% in the population, or significant deviation from Hardy–Weinberg equilibrium (HWE) in the whole sample (*p* < 0.0001) were excluded. A total number of 537,159 SNPs were included. Principal component (PC) analysis was performed to confirm that there were no samples deviating from homogeneity as a whole. The first four PCs were also included as covariates to adjust for the population stratification.

Genotype distributions were compared between the good and poor responder groups with logistic regression control using the SVS software (Golden Helix SNP and Variation Suite™ (Version 8.8.3), Bozeman, MT, USA).

### Bioinformatics analysis

Pathway-based enrichment analysis was performed using the Database for Annotation, Visualization, and Integrated Discovery (DAVID; https://david.ncifcrf.gov) to annotate the biological pathways and molecular functions of the identified genes^15^. To analyze the genes and biochemical links, the Kyoto Encyclopedia of Genes and Genomes (KEGG; https://www.kegg.jp) was used^16^.

## Result

A total of seventy-one treatment-naïve DME patients diagnosed between May 2016 and August 2017 underwent intravitreal injections. No patient was lost during follow-up. Six patients were excluded because they underwent surgery for vitreous hemorrhage, and fourteen patients were excluded due to other ophthalmic diseases identified during the follow-up period. A total of fifty-one patients were treated with intravitreal anti-VEGF injection, and twenty-five of them were classified as good responders, who did not show recurrence for more than 6 months after the last injection. Twenty-six patients were classified as poor responders. Genomic DNA extracted from whole blood in the good responder and poor responder groups were genotyped.

The demographics and clinical characteristics, including duration of DM and baseline HbA1c level, were not significantly different between the good and poor responder groups (*p* > 0.05, Table 1). Additionally, baseline CST and best-corrected visual acuity (BCVA) were not significantly different between the two groups.

**Table 1:** Demographic and clinical characteristics of the study subjects.

Characteristics		Good responder group	Poor responder group	P-value*
Total, n (female %)		25 (52)	26 (50)	.842
Age, yrs		57.4 ± 11.3	60.2 ± 9.3	.418
DM, duration, yrs		13.6 ± 11.3	13.2 ± 7.3	.082
HbA1c, %		7.23 ± 1.16	7.70 ± 1.35	.201
Hypertension, %		24.0	30.8	.291
Nephropathy, %		36.0	30.8	.445
Dyslipidemia, %		52.0	53.9	.807
Baseline	CST, μm	468 ± 126	675 ± 117	.665
	BCVA, logMAR	0.47 ± 0.33	0.49 ± 0.32	.769
After treatment at 6 month	CST, μm	257 ± 32	431 ± 98	< .001
	BCVA, logMAR	0.28 ± 0.23	0.54 ± 0.35	.003
Blood pressure, mmHg	Systolic	127 ± 9	128 ± 13	.882
	Diastolic	77 ± 7	76 ± 9	.664
BUN (Urea nitrogen), mg/dL		22.24 ± 8.73	20.13 ± 8.47	.878
Creatinine, mg/dL		1.00 ± 0.49	1.09 ± 0.61	.545
eGFR		80.5 ± 26.0	79.1 ± 24.5	.837
Cholesterol, mg/dL		153 ± 43	170 ± 41	.156
Triglyceride, mg/dL		128 ± 60	129 ± 76	.976
HDL, mg/dL		46 ± 9	52 ± 14	.124
LDL, mg/dL		87 ± 40	89 ± 33	.858

*P-values for the difference between the good and poor responder groups were obtained. DM: diabetes mellitus; CST: central subfield thickness; BCVA: best-corrected visual acuity; BUN, blood urea nitrogen; eGFR: estimated glomerular filtration rate; HDL: high-density lipoprotein; LDL: low-density lipoprotein.

### Manhattan plots and high-ranked SNPs

**Figure 2:**
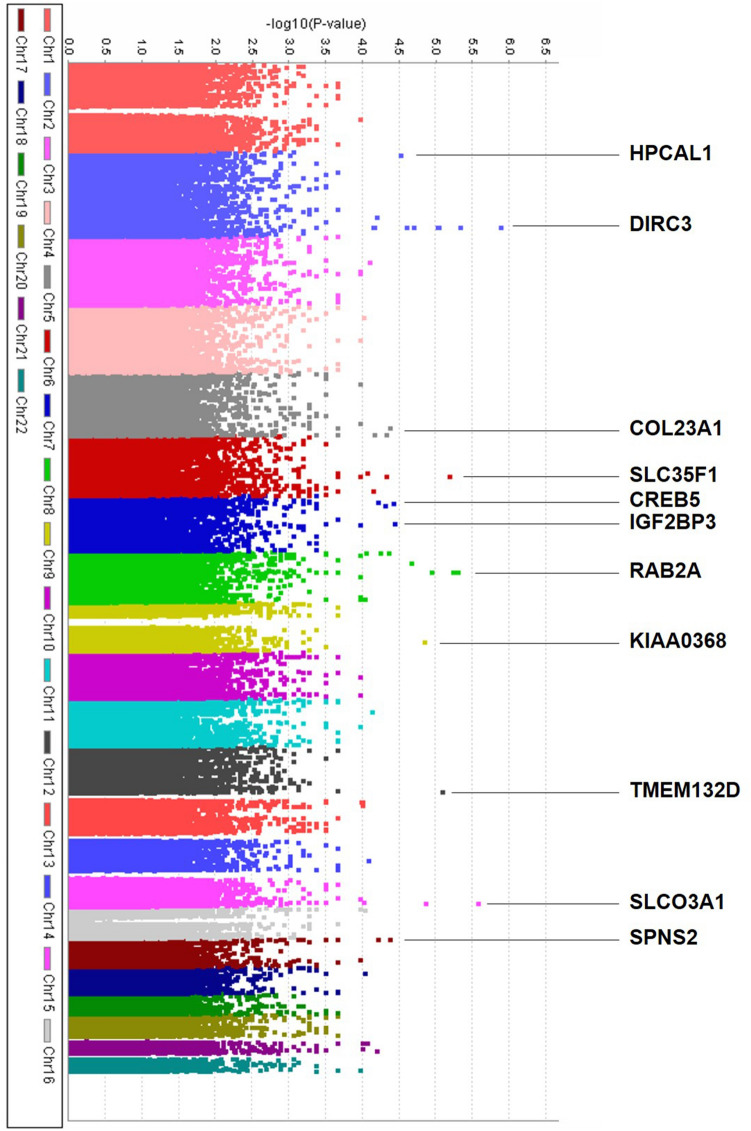
Genome-wide association analysis results of treatment response to intravitreal anti-VEGF injection to treat DME in Korean patients. Relatively significant loci are displayed with locus names (*p* < 5.0 × 10^–5^).

**Figure 3:**
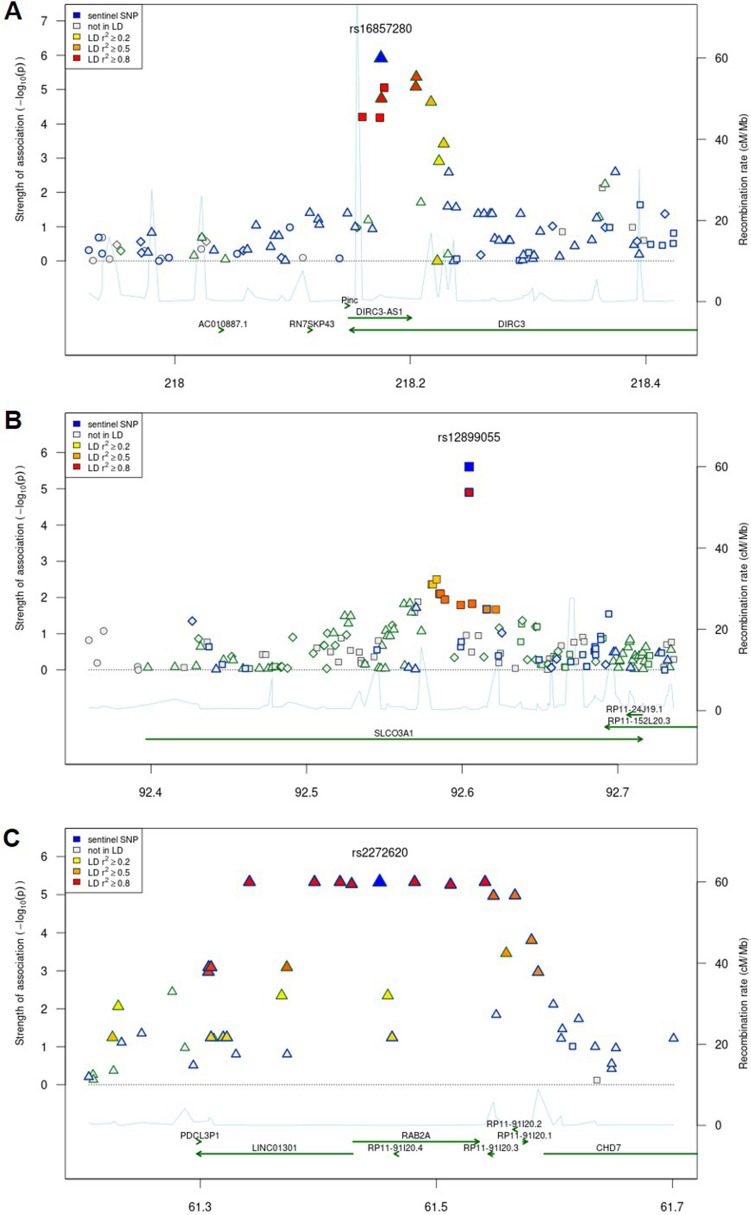
Regional association plots for three high ranked SNPs: (**A**) *DIRC3*, (**B**) *SLCO3A1*, (**C**) *RAB2A*. Lead variants are indicated by blue-filled shapes and other variants are colored according to r^2^ values with the lead variant in each SNPs.

The Manhattan plots show the GWAS results of treatment response to intravitreal anti-VEGF injection in patients with DME (Fig. 2). In the comparison between the good and poor responder groups, no SNPs reached the conventional level of genome-wide significance (*p* < 5.0 × 10^–8^), but 11 SNPs were associated with the response to intravitreal anti-VEGF injection (*p* < 5.0 × 10^–5^). The highest ranked SNP, which was located in *DIRC3* gene on chromosome 2, was associated with treatment response (rs16857280, *p* = 1.21 × 10^–6^, Fig. 3a). The minor allele frequency was higher in the good responder group (OR 18.23). On chromosome 15, *SLCO3A1* gene was related to treatment response to intravitreal anti-VEGF injection, and its minor allele was also more frequently found in the good responder group (rs12899055, *p* = 2.5 × 10^–6^, OR 23.05, Fig. 3b). Another genotyped SNP, rs2772620, located in the *RAB2A* gene on chromosome 8, was associated with treatment response. The minor allele in this SNP rs2272620 was more frequently found in the poor responder group (*p* = 4.6 × 10^–6^, OR 0.08, Fig. 3c). In addition, *SLC35F1*, *TMEM132D*, *KIAA0368*, *HPCAL1*, *IGF2BP3*, *SPN2S*, *COL23A1*, and *CREB5* genes were related to treatment response (*p* < 5.0 × 10^–5^, Table 2).

**Table 2:** Genotyped SNPs associated with treatment response to intravitreal anti-VEGF injection in patients with diabetic macular edema.

Name	Chr:Position		Gene	Allele	MAF		OR*	P-value
					Good responder group	Poor responder group		
rs16857280	2:218,175,073		DIRC3	C>T	0.440	0.071	18.23 (4.29–77.47)	1.2 × 10^–6^
rs12899055	15:92,604,530		SLCO3A1	C>T	0.640	0.268	23.05 (2.97–178.72)	2.5 × 10^–6^
rs2272620	8:61,452,046		RAB2A	G>A	0.260	0.643	0.08 (0.02–0.35)	4.6 × 10^–6^
rs11153718	6:118,372,057		SLC35F1	G>A	0.500	0.125	11.09 (3.11–39.58)	6.0 × 10^–6^
rs155370	12:130,077,096		TMEM132D	A>G	0.040	0.375	0.06 (0.01–0.31)	7.5 × 10^–6^
rs10759497	9:114,220,603		KIAA0368	G>A	0.740	0.339	8.85 (2.52–31.03)	1.3 × 10^–5^
rs11888704	2:10,530,325		HPCAL1	C>A	0.120	0.411	0.08 (0.02–0.32)	2.8 × 10^–5^
rs12700428	7:23,436,427		IGF2BP3	C>T	0.040	0.286	0.05 (0.01–0.29)	3.5 × 10^–5^
rs7213707	17:4,414,027		SPNS2	A>G	0.160	0.482	0.11 (0.03–0.37)	4.0 × 10^–5^
rs17081072	5:177,747,691		COL23A1	C>T	0.280	0.660	0.13 (0.04–0.44)	4.3 × 10^–5^
rs4722804	7:28,532,464		CREB5	G>T	0.260	0.018	31.37 (3.21–306.84)	4.5 × 10^–5^

*Odd ratios (OR) calculated with the poor responder group as a reference group and good responder group as a case group. Chr: chromosone; MAF: minor allele frequency.

### Pathway enrichment analysis

**Figure 4:**
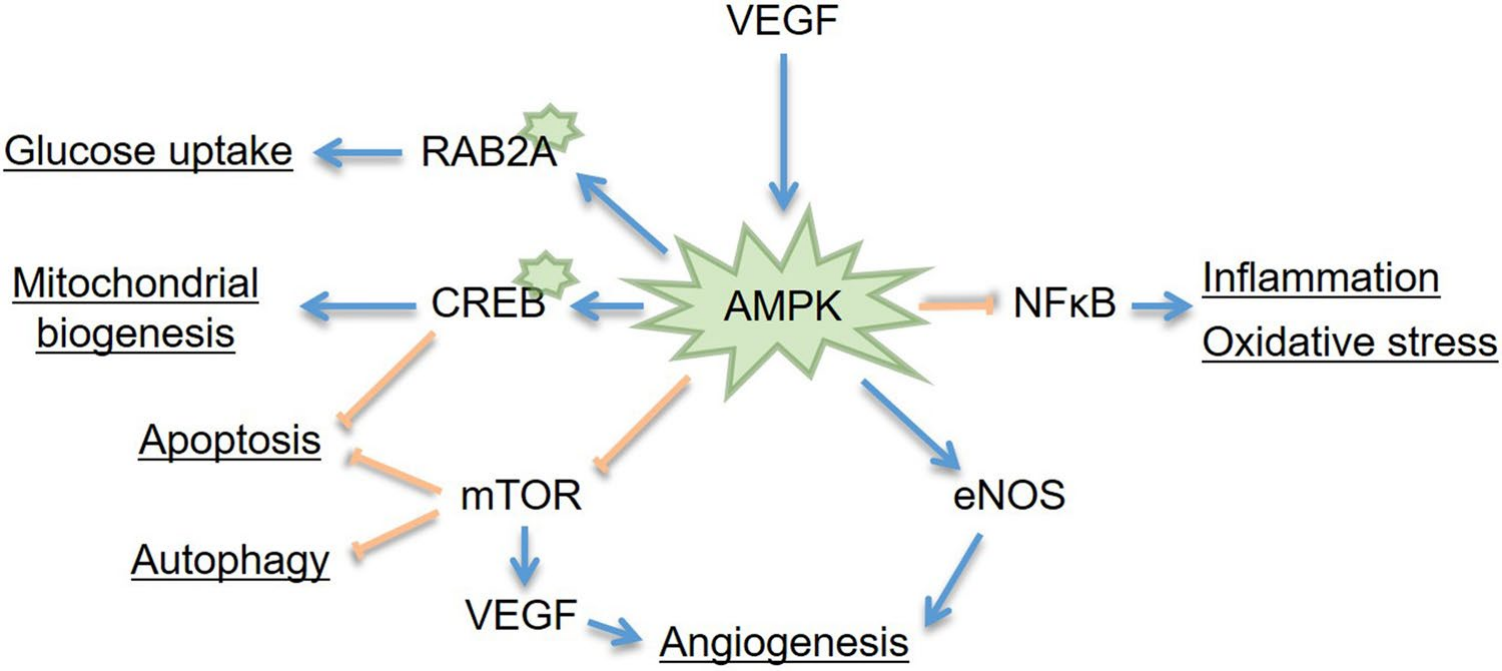
Proposed scheme for AMPK signaling pathway. *VEGF*: vascular endothelial growth factor; *AMPK*: AMP-activated protein kinase; *RAB2A*: Ras-related protein 2A; *CREB*: *CAMP* responsive element binding protein 5; mTOR: mammalian target of rapamycin; eNOS: endothelial isoform of nitric-oxide synthase; NFκB: nuclear factor kappa-light-chain-enhancer of activated B cells.

GWAS identified 11 SNPs that showed a suggestive association with response to intravitreal anti-VEGF injection (*p* < 5.0 × 10^–5^). Pathway-based enrichment analysis was performed for the genes of these SNPs using the KEGG database. The *RAB2A* and *CREB5* gene were associated with the AMP-activated protein kinase (AMPK) signaling pathway (Fig. 4, *p* = 0.018). In this signaling pathway, *RAB2A* was involved in glucose metabolism. *CREB5* was found to be related to mitochondrial biogenesis and apoptosis in the AMPK signaling pathway.

## Discussion

To our knowledge, this is the first GWAS reported in relation to the treatment response of DME. In this study, we analyzed the relationship between genetic profiles and treatment outcomes of intravitreal anti-VEGF injection of DME in Korean patients. Eleven SNPs were associated with the treatment response. From the enriched pathway analysis based on KEGG, these genes were found to be related to the AMPK signaling pathway.

There are studies reporting the expression of genes associated with treatment response in DME, but these did not utilize GWAS. Shazly et al. reported a significant correlation between the genetic characteristics and response to intravitreal anti-VEGF (bevacizumab) in patients with DME. The distribution and allele frequency of the VEGFA -634C>G polymorphism (rs2010963) were significantly higher in good responders than in poor responders (*p* < 0.001)^17^. Tetikoglu et al. studied the association between VEGFA gene polymorphisms and response to intravitreal anti-VEGF (ranibizumab) injection in patients with DME, but no significant association was found^18^. In a pilot study that performed GWAS to differentiate between bevacizumab responders and non-responders, the majority of expressed genes were in transcriptional regulation or receptor activation pathways^19^. A recent study in a Middle Eastern population investigated the relationship between serum hyperglycemia-related long noncoding ribonucleic acid (lncRNA) levels and response to anti-VEGF (aflibercept) injection, but no significant association was found^20,21^. To date, only one recent report has conducted a GWAS to identify genes associated with the response to anti-VEGF treatment in DME, which has been performed in the Australian population^22^. In this report, the treatment response was determined based on the change in CST 12 months after the initiation of anti-VEGF injections, and several significant loci were identified. However, to date, no such GWAS has been performed in the Asian population.

The highest-ranked SNP rs16857280 is located in *DIRC3*, which was associated with treatment response. Diseases associated with *DIRC3* include renal cell carcinoma, breast cancer, and skin cancer^23^. In a recent study, *DIRC3* was found to act as a tumor suppressor, blocking the growth of human melanoma, and patients with high levels of *DIRC3* showed significantly improved survival rates compared to those with low level ^24^. It has been shown that *DIRC3* can be used to identify novel targets for skin cancer treatment. In this study, *SLCO3A1* (rs12899055) was related to treatment response, and its minor allele was more frequently found in the good responder group (OR 23.05). In another GWAS, *SLCO3A1* (rs207959) mediated inflammatory processes through the nuclear factor kappa-light-chain-enhancer of activated B cells (NFκB) in Crohn's disease^25^. These genes have not yet been identified as being related to DR or DME. Because these genes were shown to be related to treatment response in this study, the effect and associated mechanism of action on anti-VEGF treatment should also be taken into consideration.

rs2272620 is located in the *RAB2A* gene. *RAB2A* is known to be involved in glucose metabolism via the AMPK signaling pathway. AMPK translocates GLUT4 from intracellular storage vesicles to the plasma membrane, increasing glucose uptake into cells. In this process, translocation and fusion of GLUT4 vesicles are regulated by the Rab family, which is activated by AMPK^26^. *RAB2A* is also associated with cellular damage and apoptosis in Müller cells^27^. Müller cells are the primary glial cells in the retinal tissue that perform a wide range of functions, including maintaining the blood-retinal barrier. Hyperglycemia causes Müller cell dysfunction and loss, associated with *RAB2A*^28^. As *RAB2A* was associated with anti-VEGF treatment response, it may be the AMPK signaling pathway or Müller cell dysfunction in the VEGF-related pathways.

AMPK modulates cellular metabolism in response to stress, such as malnutrition, hypoxia, or exercise^29^. Pathway-based enrichment analysis using the KEGG database revealed that the AMPK signaling pathway was associated with the *RAB2A* and *CREB5* genes (*p* < 0.05). Activation of AMPK in endothelial cells by VEGF represents a pro-angiogenic pathway^30^. Intracellular metabolic states are involved in the regulation of angiogenesis, which may involve AMPK. In addition to the glucose metabolism pathways in which *RAB2A* is involved, AMPK has a protective role in retinopathy^31^. AMPK promotes angiogenesis by increasing the production of VEGF and the endothelial isoform of nitric-oxide synthase (eNOS), which dilates retinal arterioles to improve circulation^32^. AMPK may attenuate angiogenesis by inhibiting mammalian target of rapamycin (mTOR) signaling as tumor-promoting and pro-metastatic factors^33^. Although the mechanisms are not clearly understood, they protect retinal neurons, glial cells, and retinal pigment epithelial cells by inhibiting oxidative stress and inflammation in the AMPK signaling pathway^34^. AMPK signaling inhibits the inflammatory response induced by NF-κB. Another study reported that *SLCO3A1*, which was identified in this study, was also associated with NFκB in regulating inflammation^35^. In a previous study that performed miRNA profiling and cytokine assays of the aqueous humor of DME patients, the aqueous concentrations of not only VEGF, but also inflammatory cytokines such as interleukin-6 and 8, were significantly higher in the DME group compared to the control group^36^. Inflammation or other factors may play an important role in the pathogenesis of DME and can be potential molecular targets for anti-VEGF injection and future DME treatment.

There are some limitations to this study that may have influenced the conclusions. First, the data were obtained from only one ethnic group (Korean) and may not be applicable to other ethnic groups. To generalize these results, data on genetic characteristics from different ethnic groups should be obtained and analyzed based on clinical characteristics. Second, due to the small sample size, no SNPs reached genome-wide significance (5e−8). Instead, we identified several loci with *p* values suggestive of association (5e−5). Third, there was also a lack of comparison with normal controls or patients with other disease or treatment response according to the drug. Further analysis may be helpful for initiating intravitreal injection therapy in patients with naive DME. In addition, analysis of the treatment response according to the subtypes of DME (according to morphology) or medication history could have provided meaningful information; however, this was not feasible due to the small sample size (of each subtype). Future studies with larger sample sizes and analyses based on subtypes are needed.

This study is the first to report a GWAS on the treatment response of Korean patients with DME to intravitreal anti-VEGF injection (the primary treatment). This study may allow ophthalmologists to personalize treatment options based on the patient’s genetic profile, improve drug selection, and reduce trial and error. In addition, it will help researchers understand the pathways related to the pathogenesis and treatment of DME.

## Data Availability

The datasets generated and analysed during the current study are available in ClinVar, https://www.ncbi.nlm.nih.gov/clinvar/submitters/508569.
